# EHMTI-0095. Spreading depression enhances neurogenesis in hippocampus and dentate gyrus

**DOI:** 10.1186/1129-2377-15-S1-F15

**Published:** 2014-09-18

**Authors:** M Lotfinia

**Affiliations:** 1 Neuroscience, Shefa Neuroscience Research Center, Tehran, Iran

## Introduction

Spreading depression (SD) known by transient loss of spontaneous and evoked neuronal activity and changes in ionic, metabolic and hemodynamic characteristics of the brain. Neuronal damage followed by SD, supposed to have a dramatic impression on SD-derived pathologic conditions. We aimed to determine whether SD is able to stimulate persistent neurogenesis.

## Methods

Wistar rat (60-80gr) randomly chosen and 3 mol/L KCl injected for induction of SD. Four weeks after the first injection, all rats were decapitated and the brains removed. The density of mitotic cells, divided cells, and new neurons in the pyramidal cell layer of hippocampal CA1 and CA3 and granular cell layer of dentate gyrus was assessed. We also detect the DNA during the S phase using Bromodeoxyuridine (BrdU).

## Results

A remarkable increase occurred in the number of BrdU-labeled cells in hippocampal region, detected by immunohistochemistry method. The density of mitotic cells, divided cells, and new neurons in hippocampal CA1 and CA3 and granular cell layer of dentate gyrus also increased.

## Conclusion

We conclude that Spreading depression potentiates to trigger persistent neurogenesis in rat hippocampus.

No conflict of interest.

